# Ion channels in somatosensory transmission: an introduction to the collection

**DOI:** 10.12688/f1000research.5729.1

**Published:** 2014-11-14

**Authors:** Nikita Gamper

**Affiliations:** 1 School of Biomedical Sciences, Faculty of Biological Sciences, University of Leeds, Leeds, UK

## Abstract

Excitation of peripheral endings of sensory nerves is a primary event in most types of somatosensation, including pain. This excitation and transmission of action potentials within somatosensory pathways is brought about by the concerted action of the wide array of plasmalemmal ion channels, some of which are specific to somatosensory nerves. Accordingly, ion channel deficiencies or ‘channelopathies’ often underlie sensory disorders and pathological pain states and many current and prospective analgesics target ion channels. This *F1000Research* article collection is focused on the current advances in understanding function and regulation of ion channels controlling excitability and synaptic transmission within somatosensory pathways. The focus is on the peripheral neurons but studies of central mechanisms that integrate peripheral inputs are also welcome. We also welcome discussions of emerging approaches, methods and techniques in somatosensory physiology.

## Editorial

Peripheral somatosensory neurons underlie a variety of somatic sensations, which can be torturing or pleasant. These neurons are different from most other neurons in the mammalian nervous system in several key features. Thus, these neurons have a very specific anatomy, being pseudo-unipolar neurons with a single giant neuritis that are split in a t-way and can reach several meters in length in some mammals. The action potentials in the somatosensory neurons normally originate not at the axon initial segment (as in most CNS neurons) but at the nerve endings within the peripheral tissues (skin, muscles, joints, blood vessels, internal organs etc.); therefore, the giant neurites (or fibres) of the somatosensory neurons serve both axonic and dendritic functions. Major segments of a somatosensory neuron (that is the cell body, axonal stem, peripheral and most of the central branches of the fibre) are located outside the blood-brain barrier and, thus, are exposed to circulation at least to some extent. Yet, the central branch of the fiber enters the CNS as it synapses in the dorsal horn of the spinal cord. Thus, the peripheral somatosensory neuron in fact belongs to both peripheral and central nervous systems simultaneously. There are other important distinctions of peripheral somatosensory neurons, for instance they accumulate high intracellular chloride levels so that, in contrast to the CNS neurons, activation of chloride channels in these neurons can result in excitation instead of inhibition ( Liu *et al.*, 2010). Finally, these neurons express a specific set of ion channels, some of which are more or less unique to this type of neurons ( Raouf *et al.*, 2010), such as a voltage gated Na ^+^ channel Nav1.7 for example.

Recent decades have seen a tremendous advance in our understanding of mechanisms of peripheral sensory neuron excitability and how these are different from CNS neurons; specific subpopulations of sensory neurons that respond to particular stimuli (e.g. mechanical, thermal, chemical) and underlie specific sensations (e.g. touch, pain, itch) have been identified ( Basbaum *et al.*, 2009); many sensory neuron-specific ion channels have been cloned and characterised ( Raouf *et al.*, 2010); and mechanisms of many sensory dysfunctions, leading, for instance, to chronic pains have been revealed ( McMahon *et al.*, 2006). Yet, new channels are being cloned and new mechanisms are being characterized every year and there is still huge uncharted territory to discover. Thus, despite the cloning of Piezo channels ( Coste *et al.*, 2010), the molecular mechanisms of mechanotrasduction are still not completely understood; mechanisms behind many basic phenomena such as noxious cold sensation or inflammatory hyperalgesia are intensely debated. Most importantly, despite decades of research and investment, there is still little progress in analgesic drug development and opioids are still a gold standard.

This *F1000Research* article collection is focused on the current advances in understanding function and regulation of ion channels controlling excitability and synaptic transmission within somatosensory pathways. The focus is on the peripheral neurons but studies of central mechanisms that integrate peripheral inputs are also welcome. We also welcome discussions of emerging approaches, methods and techniques in somatosensory physiology.

## References

[ref-1] Basbaum AI Bautista DM Scherrer G: Cellular and molecular mechanisms of pain. *Cell.* 2009;139(2):267–284. 10.1016/j.cell.2009.09.028 19837031 PMC2852643

[ref-2] Coste B Mathur J Schmidt M: Piezo1 and Piezo2 are essential components of distinct mechanically activated cation channels. *Science.* 2010;330(6000):55–60. 10.1126/science.1193270 20813920 PMC3062430

[ref-3] Liu B Linley JE Du X: The acute nociceptive signals induced by bradykinin in rat sensory neurons are mediated by inhibition of M-type K ^+^ channels and activation of Ca ^2+^-activated Cl ^-^ channels. *J Clin Invest.* 2010;120(4):1240–1252. 10.1172/JCI41084 20335661 PMC2846053

[ref-4] McMahon SB Koltzenburg M Wall PD: Wall and Melzack’s textbook of pain. Edinburgh: Elsevier Churchill Livingstone.2006 Reference Source

[ref-5] Raouf R Quick K Wood JN: Pain as a channelopathy. *J Clin Invest.* 2010;120(11):3745–3752. 10.1172/JCI43158 21041956 PMC2965577

